# Disseminated Hydatid Disease With Extensive Abdominal and Pelvic Involvement In a Young Male: A Case Report

**DOI:** 10.31729/jnma.v63i290.9195

**Published:** 2025-09-01

**Authors:** Kritendra Raj sharma, Niranjan Palikhe, Sunil Shrestha, Rahul Mahaseth, Baidh Gupta, Rupesh Sah

**Affiliations:** 1 Department of Surgery, Bluecross Hospital, Lalitpur, Nepal; 2 Department of Surgery, Star Hospital Limited, Lalitpur, Nepal; 3 Department of General and GI Surgery, Star Hospital Limited, Sanepa Heights, Lalitpur, Nepal; 4 Health Service Office, Jajarkot, Nepal; 5 Department of Medicine, P.T. Birta City Hospital, Birtamod, Nepal; 6 Evergreen Lifecare Hospital, Mahjidhiya, Lumbini, Nepal

**Keywords:** *albendazole*, *disseminated hydatidosis*, *Echinococcus granulosus*, *hydatid disease*, *Nepal*

## Abstract

Hydatid disease, a zoonotic infection caused by Echinococcus granulosus, typically involves the liver and lungs. Disseminated abdominal hydatidosis is rare and often results from cyst rupture or inadequate treatment. A 27-year-old male from rural Nepal presented with abdominal pain, jaundice, and palpable masses. Imaging revealed disseminated hydatid cysts in the liver, peritoneum, and pelvis. He had received albendazole monotherapy abroad. Surgical removal of the cysts was performed, followed by albendazole therapy. This case emphasizes the need for early diagnosis, complete management including surgery and antiparasitic therapy, and awareness of dissemination risks due to incomplete treatment.

## INTRODUCTION

Hydatid disease is a parasitic infection primarily caused by the larval stage of Echinococcus granulosus, with E. multilocularis being a rarer but more aggressive form.^1,2^ Dogs and other canids are definitive hosts, while livestock serve as intermediate hosts. Humans become accidental hosts by ingesting eggs through contact with infected animals or contaminated food and water.^3,4^ The liver is the most commonly affected organ, followed by the lungs and, less often, the spleen or kidneys.^5,6^ In rural Nepal, close human-animal contact sustains endemic transmission.^7^ Disseminated disease is rare, often resulting from cyst rupture and may require surgical intervention.^8^

## CASE REPORT

A 27-year-old male, originally from Myagdi district, Nepal, presented with a three-year history of progressive abdominal pain and distension. He had no significant past medical history. He had been working in Malaysia for the past three years, where his symptoms initially began.

The patient first experienced vague abdominal discomfort and progressive distension approximately three years ago while residing in Malaysia. He was evaluated at a local hospital where a CT scan revealed multiple intra-abdominal cystic lesions, free fluid in the abdomen and pelvis and a diagnosis of disseminated hydatid disease was made. He was started on oral Albendazole 200 mg once daily for six months. However, within two to three weeks of starting therapy, his symptoms worsened, with increasing abdominal distension, anorexia, nausea, jaundice, and urinary discomfort. Due to this symptom progression, he returned to Nepal and presented to our center for further evaluation and management.

At presentation, the patient was afebrile, with a visibly distended abdomen and generalized tenderness. There was no hepatomegaly. He denied vomiting, diarrhea, fever, or rash. He did report respiratory discomfort, though cardiovascular findings were within normal limits.

Laboratory investigations revealed a total leukocyte count of 10,700/mm^3^ (63% neutrophils, 16% eosinophils), with an elevated erythrocyte sedimentation rate of 54 mm/hr. Liver enzymes were mildly elevated (ALT 56.3 IU/L, AST 57 IU/L, ALP 318 IU/L). Serum total protein was 7.7 g/dl, and albumin was slightly reduced to 3.5 g/dl. Viral serologies for hepatitis C and HIV were negative. A plain abdominal X-ray was unremarkable.

**Figure 1:**
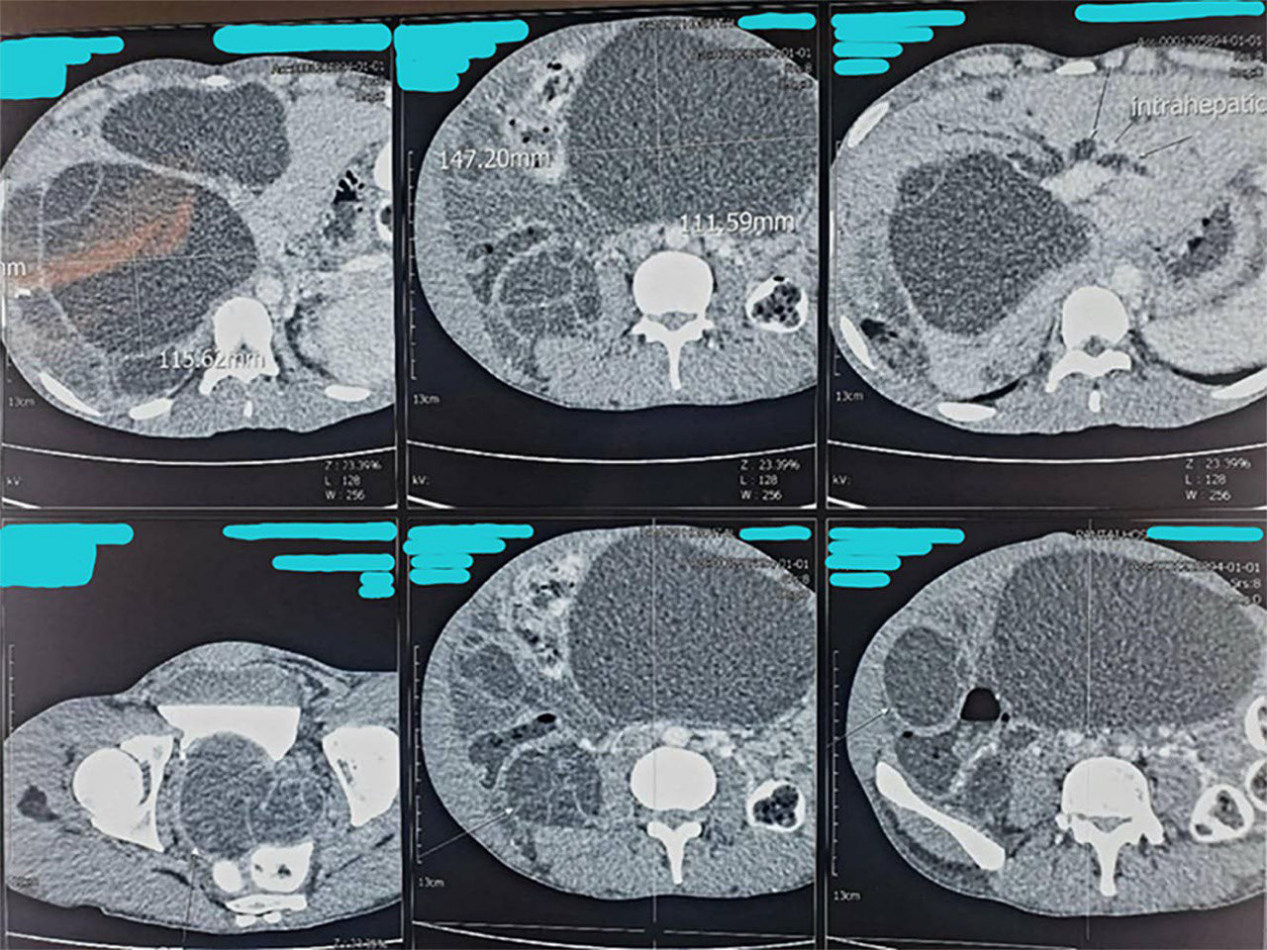
Contrast-enhanced CT scan of the abdomen showing multiple upper axial sections of the liver with a large intrahepatic hydatid cyst. The cyst measures approximately 111.59 mm × 147.20 mm at its maximal dimensions. The slices demonstrate the extent of the cyst within the liver parenchyma.

Given the clinical findings and deranged liver enzymes, an abdominal ultrasound was performed. It showed multiple cystic lesions with internal septations and daughter cysts within the liver parenchyma. The largest lesion, measuring 10.1 × 8.6 cm, was located in the right lobe. Additional cysts were noted in the abdominal and pelvic cavities, the largest in the pelvic cavity measuring 7.5 × 6.0 cm. The imaging findings were consistent with hydatid cysts. The patient also brought the CT scan previously performed in Malaysia, which revealed numerous cystic lesions with enhancing septations and daughter cysts in both hepatic lobes—the largest measuring 11.5 × 13.3 × 12.2 cm in the right lobe (Figure 1).

**Figure 2:**
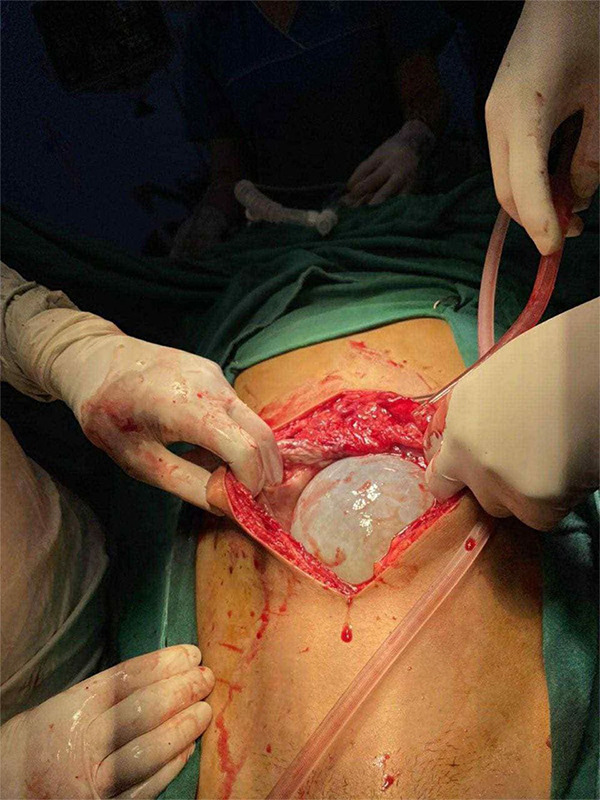
Intraoperative photograph showing a large hydatid cyst emerging through a midline laparotomy incision.

**Figure 3:**
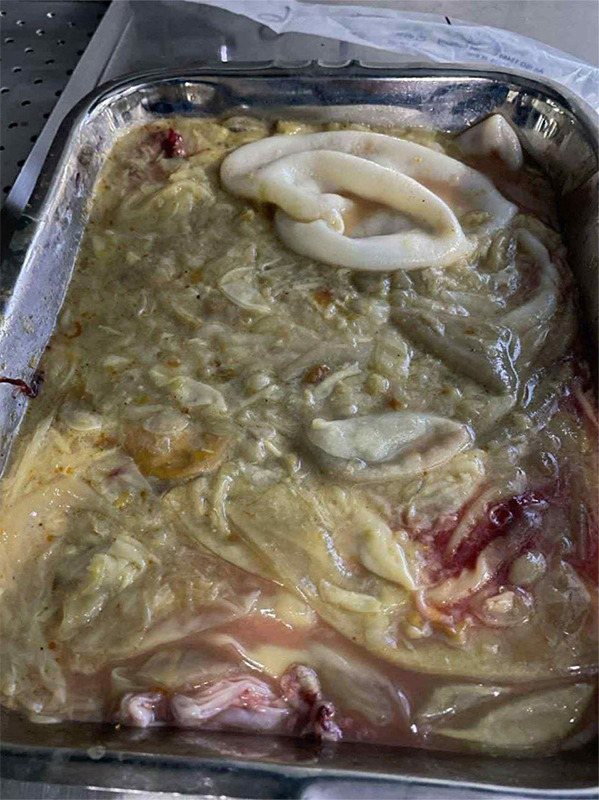
Gross specimen photograph showing multiple excised hydatid cysts along with pericystic tissues and omental adhesions collected intraoperatively. The cysts vary in size, consistent with disseminated hydatid disease involving the liver and peritoneum.

**Figure 4:**
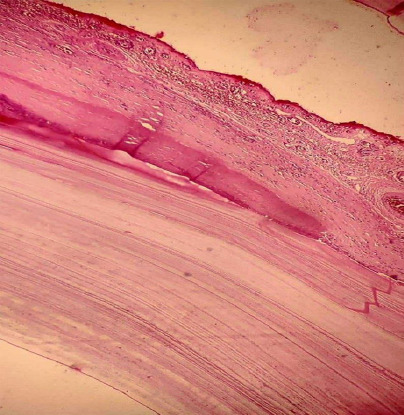
Photomicrograph of a hydatid cyst wall showing its trilaminar structure. The image demonstrates the outer fibrous pericyst (host reaction), middle laminated acellular ectocyst, and inner germinal layer. The section is stained with Hematoxylin and Eosin (H&E).

Similar lesions were seen in the abdomen and pelvis, including a large cyst in the mid-abdomen measuring 11.1 × 14.7 × 17.7 cm. Free intraperitoneal fluid was also noted.

Based on his clinical history, imaging, prior diagnosis, and endemic exposure, a diagnosis of disseminated hydatid disease was confirmed. Due to the extensive involvement, a surgical approach was planned. He was started on oral albendazole 400 mg twice daily for three days as preoperative therapy before undergoing surgery. A midline laparotomy was performed revealing multiple hydatid cysts involving the liver and peritoneal cavity (Figure 2). The excised specimen, comprising multiple hydatid cysts with associated pericystic tissue and omental adhesions (Figure 3). Histopathological examination of the cyst confirmed the diagnosis (Figure 4).

The patient had an uneventful postoperative recovery. Albendazole therapy was continued after surgery. He was discharged in stable condition and advised for regular outpatient follow-up.

## DISCUSSION

Hydatid disease, caused by the larval form of Echinococcus granulosus, primarily affects the liver (75%) and lungs (15%), owing to their roles as the first and second filters in the portal and systemic circulations, respectively.^1,2^ However, disseminated echinococcosis, such as peritoneal and mesenteric involvement, is rare and typically results from rupture of hepatic cysts — either spontaneously, post-trauma, or after incomplete medical or surgical intervention.^9^

In our case, the patient had a history of partial medical therapy and later presented with intraabdominal dissemination without prior surgery or trauma. This suggests possible secondary echinococcosis resulting from undiagnosed cyst rupture or inadequate sterilization of cysts by albendazole alone. Incomplete or unsupervised therapy has been associated with cyst survival and progression to dissemination.

Diagnosis of hydatid disease typically starts with imaging, but serological immunoassays such as enzyme-linked immunosorbent assay (ELISA) and indirect hemagglutination (IHA) provide important adjunctive value. ELISA is commonly used due to high sensitivity (85-98%) and specificity (88-96%) for hepatic cysts.^10,11^

Ultrasonography (USG) is the first-line imaging modality, especially useful in endemic areas due to its availability and ability to classify cyst types (WHO CE classification). The WHO Informal Working Group on Echinococcosis classifies hepatic hydatid cysts into six types based on ultrasonographic appearance: CE1 (simple active cyst), CE2 (multivesicular active), CE3a (transitional with detached membrane), CE3b (daughter cysts in solid matrix), CE4 (degenerating inactive), and CE5 (calcified inactive).^12^ Preoperative USG is also crucial for surgical planning as it helps assess cyst wall viability, presence of daughter cysts, biliary communication, and risk of rupture. In addition, Doppler USG can help in vascular mapping and differentiating cysts from abscesses or tumors.

CT and MRI provide superior spatial resolution, especially for detecting calcified, multiorgan, or extrahepatic disease. In our case, CT imaging played a pivotal role in defining the extent of dissemination and in differentiating cysts from abscesses or neoplastic masses.^13^

Treatment of hydatid disease involves a multimodal approach. Albendazole is the drug of choice, especially in early, small, or multiple inoperable cysts. However, in large or complicated cysts, surgical excision remains the definitive therapy. In our case, surgery successfully relieved the patient’s symptoms, with the procedure effectively reducing the parasitic load.^14^

PAIR (Puncture-Aspiration-Injection-Reaspiration) is a minimally invasive technique with success in treating liver hydatids (WHO types CE1 and CE3a). However, PAIR is contraindicated in disseminated hydatidosis due to the high risk of leakage, secondary spread, and anaphylaxis from multiple punctures.^5^ Moreover, PAIR is not ideal when cysts are located near vital structures, heavily calcified, or communicate with biliary or peritoneal spaces.

Spillage of protoscolices from ruptured cysts can cause peritoneal seeding and form multiple secondary cysts. Albendazole pretreatment for at least 1 month preoperatively and continued for 1-3 months postoperatively has been shown to reduce recurrence risk by sterilizing cysts and minimizing viable scolices.^15^ In this case, the patient had an uneventful postoperative recovery, with albendazole therapy continued after surgery.

Though Nepal is considered low prevalence for hydatidosis, cases do occur, often in patients with a rural background or history of livestock exposure. Underreporting, misdiagnosis, and limited rural imaging capabilities might mask the true burden. Our case underscores the importance of early diagnosis, strict medical supervision, and long-term follow-up in suspected hydatidosis.

## CONCLUSION

Disseminated hydatid disease, though rare, should be considered in patients with chronic abdominal distension and cystic lesions from endemic areas like Nepal. Early diagnosis is crucial to prevent complications such as cyst rupture. Incomplete treatment may lead to disease progression and necessitate surgery. Accurate diagnosis requires comprehensive imaging and serology. Surgical intervention is preferred for large or complicated cysts, while medical therapy and PAIR suit selected cases. Albendazole, given pre- and post-operatively, helps minimize recurrence and improve outcomes.
